# Functional diversity and coexistence of herbaceous plants in wet, species‐rich savannas

**DOI:** 10.1002/ece3.7404

**Published:** 2021-03-22

**Authors:** John Stephen Brewer, Peter Zee

**Affiliations:** ^1^ Department of Biology University of Mississippi University MS USA

**Keywords:** adaptation to fire, competition, encroachment, fire exclusion, functional traits, natural experiment, niche, rarefaction

## Abstract

Trait differences among plant species can favor species coexistence. The role that such differences play in the assembly of diverse plant communities maintained by frequent fires remains unresolved. This lack of resolution results in part from the possibility that species with similar traits may coexist because none has a significant fitness advantage and in part from the difficulty of experimental manipulation of highly diverse assemblages dominated by perennial species. We examined a 65‐year chronosequence of losses of herbaceous species following fire suppression (and subsequent encroachment by *Pinus elliottii*) in three wet longleaf pine savannas. We used cluster analysis, similarity profile permutation tests, and k‐R cluster analysis to identify statistically significant functional groups. We then used randomization tests to determine if the absence of functional groups near pines was greater (or less) than expected by chance. We also tested whether tolerant and sensitive species were less (or more) likely to co‐occur by chance in areas in savannas away from pines in accordance with predictions of modern coexistence theory. Functional group richness near pines was lower than expected from random species extirpations. Wetland perennials with thick rhizomes and high leaf water content, spring‐flowering wetland forbs (including *Drosera tracyi*), orchids, *Polygala* spp., and club mosses were more likely to be absent near pines than expected by chance. C3 grasses and sedges with seed banks and tall, fall‐flowering C4 grasses were less likely to be absent near pines than expected by chance. Species sensitive to pine encroachment were more likely to co‐occur with other such species away from pines at two of the three sites. Results suggest that herb species diversity in frequently burned wet savannas is maintained in part by a weak fitness (e.g., competitive) hierarchy among herbs, and not as a result of trait differences among co‐occurring species.

## Graphical abstract

The role that trait differences play in the assembly of diverse plant communities maintained by frequent fires remains unresolved. A 65‐year chronosequence of losses of herbaceous species following fire suppression in three wet pine savannas revealed that functional diversity near pines was lower than expected from random species extirpations. However, herb species diversity appeared to be maintained in part by a weak fitness hierarchy among herbs and not as a result of trait differences among co‐occurring species.

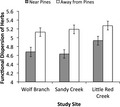

## 1 INTRODUCTION

The role that trait differences play in the assembly of diverse plant communities maintained by frequent, low‐intensity disturbances is unclear. Chronic low‐intensity disturbances such as fire, mowing, or grazing prevent competitive displacement of small herbs by larger herbs and woody plants (Brewer, 2017; Klimeš et al., 2001; Myers & Harms, 2009; Wilson et al., 2012). Such hyper‐diverse assemblages appear to support hypotheses of species coexistence maintained by disturbance (e.g., Huston, 1979), fitness similarity, and dispersal limitation (Hubbell, 2001; Myers & Harms, 2009), but several ecologists have argued that stabilizing niche differences among species are necessary to explain the maintenance of species diversity over the long term, even when fitness similarity, disturbances, and dispersal limitation are sufficient to maintain short‐term species coexistence (Adler et al., 2010; Chesson, 2000; Fox, 2013; Levine & HilleRisLambers, 2009).

Because experimental tests of the effects of niche differences on species coexistence are prohibitive for diverse assemblages dominated by long‐lived perennials at the community level, examination of spatial patterns of multiple functional traits has been offered as a way to test such niche effects on species coexistence (Kraft et al., 2008; McGill et al., 2006). To the extent that functional trait differences between species represent niche differences, functional trait dispersion could be used as a proxy or indicator of niche differences among co‐occurring species (Kraft et al., 2015). Alternatively, interspecific differences in functional traits may represent differences in fitness rather than stabilizing niche differences, in which case coexistence may result from functional similarities among co‐occurring species (Levine & HilleRisLambers, 2009) and intraspecific trait variation (e.g., adaptive phenotypic plasticity) in response to interspecific competition (Brewer, 2003; 2019; Bennett et al., 2016, Carmona et al., 2019).

**FIGURE 1:**
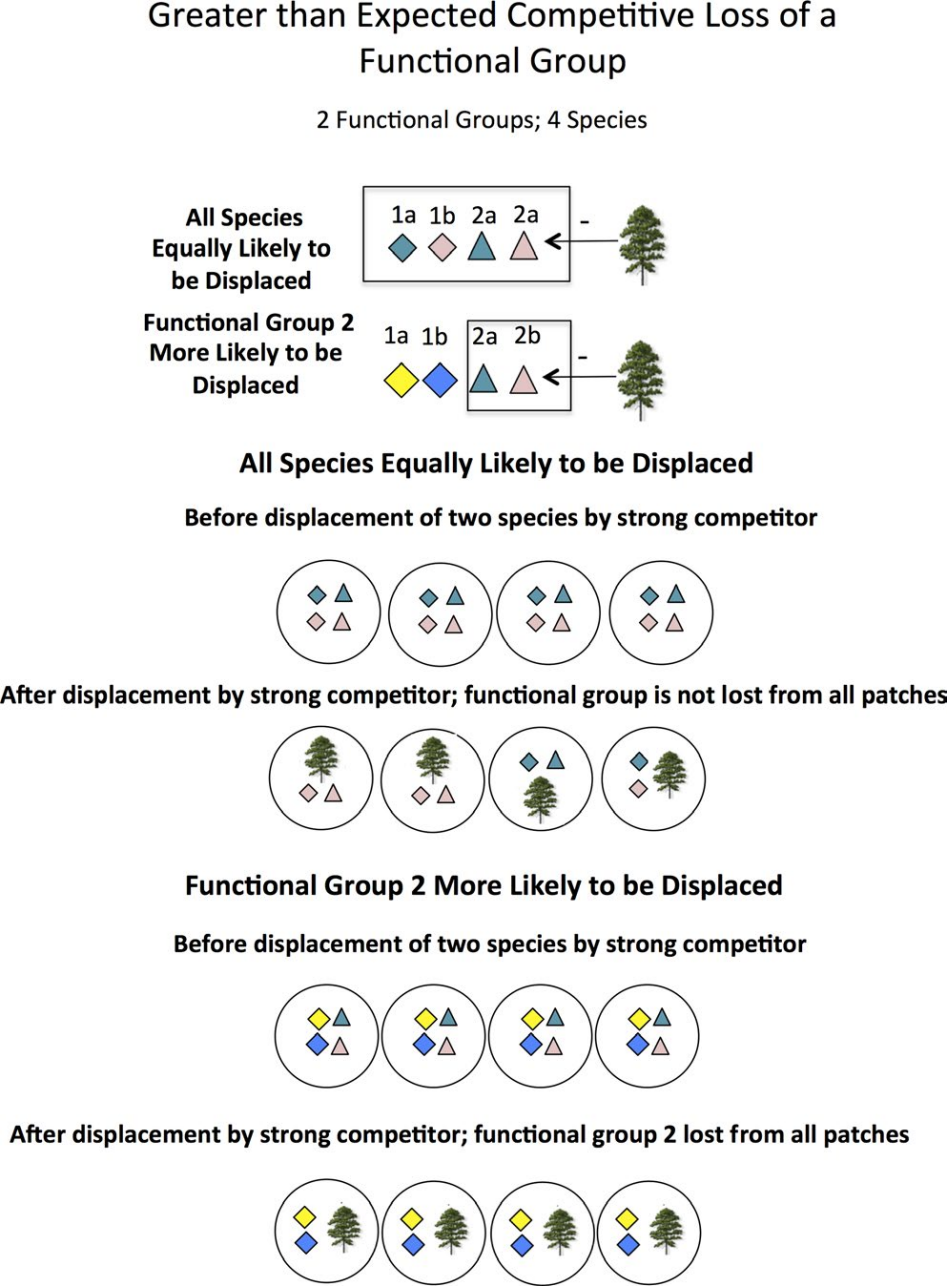
Contrast of the effects of two species being displaced by a strong competitor when species loss is random versus when species of a particular functional group are more likely to be displaced than others

**FIGURE 2:**
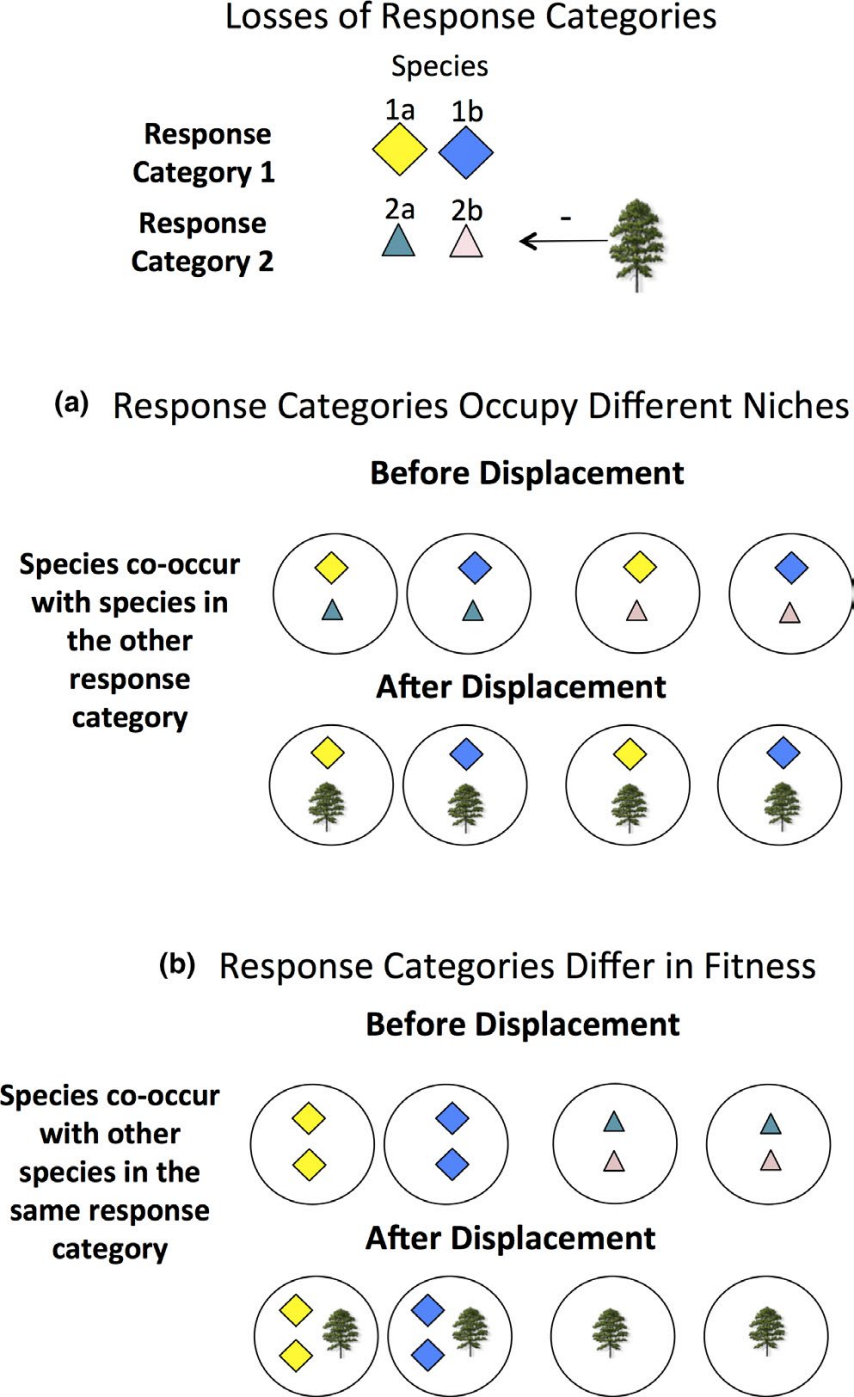
Predicted effects of two species being displaced by a strong competitor when species of different response categories (a) are more likely to co‐occur due to niche differences and (b) are less likely to co‐occur due to differences in competitive ability, consistent with alternative hypotheses of modern species coexistence theory (Levine & HilleRisLambers, 2009; Kraft el., 2015)

A potentially promising approach to understanding competition and species coexistence in diverse assemblages of long‐lived plants is to examine the long‐term pattern of losses of functional groups following the natural addition of a strong competitor (Brewer, 2017). If the disassembly of an assemblage following the addition of such a competitor is not random, then it is likely that community assembly prior to the addition of the competitor was not random either (Brewer, 2017; Myers & Harms, 2009, 2011). When species in an assemblage are represented by a relatively large number of functional groups that vary in their response to a strong competitor, losses of functional groups can be greater than expected from random species losses (Figure 1). Such losses could occur when the added competitor occupies a similar niche to that of species within some functional groups but not others (Figure 2a). Prior to the addition of a strong competitor, species with high fitness (i.e., those tolerant of competition from the strong competitor, Response Category 1; Figure 2) could co‐occur with less fit species (i.e., species sensitive to competition, Response Category 2; Figure 2) because of niche (functional) differences between these species. Consequently, species that differ in fitness and thus in their response to the strong competitor should co‐occur more often than expected by chance prior to the addition of the strong competitor (Figure 2a). Alternatively, if species co‐occurrence results from species being more‐or‐less equal in fitness prior to the addition of strong competitor, then species within the same response category should be more likely to co‐occur than expected by chance (Figure 2b).

In species‐rich, wet longleaf pine (*Pinus palustris* Mill.) savannas of the southeastern USA, herbaceous plants are hypothesized to be vulnerable to displacement by woody plants, including pines (Brewer, 1998). The specific mechanisms driving the losses of herbaceous species remain poorly understood, but could involve the reduction of soil moisture levels through hydraulic conductance (Gonzalez‐Benecke et al., 2011), pine litter deposition (Brewer et al., 2018), and/or disruption of mutualisms or facilitation (apparent competition) (Becklin et al., 2012), or intransitive effects. What is clear is that prolonged fire exclusion results in increased woody plant abundance and cover and concomitant declines in herb species diversity (Ames et al., 2017; Brewer, 2017; Brewer et al., 2018; Brockway & Lewis, 1997; Glitzenstein et al., 2003, 2012; Hinman & Brewer, 2007; Myers & Harms, 2009; Palmquist et al., 2014). In regard to wet pine savannas along the coast of the Gulf of Mexico in Mississippi (USA), the abundance and diversity of herbs decline following encroachment and increased densities of off‐site pines such as slash pine (*Pinus elliottii*) during prolong periods of fire exclusion (Brewer, 2017; Brewer et al., 2018).

The processes responsible for herb species coexistence in wet pine savannas have not been elucidated (Brewer, 2003). Previous analyses of functional traits in pine savannas have indicated that species rarity is driven by fire exclusion and that rare species exhibit different traits than common species (Ames et al., 2017). Precisely what makes certain functional groups vulnerable to fire exclusion, however, is not clear. In wet pine savannas in southern Mississippi, wetland forbs (e.g., carnivorous plants and small forbs with well‐developed root aerenchyma and high leaf moisture) may co‐occur with mesophytic grasses (e.g., *Muhlenbergia expansa* (Poir.) Trin., *Ctenium aromaticum* (Walter) Alph. Wood) (Brewer et al., 2011). If these wetland forbs and mesophytic grasses occupy different niches, then niche theory predicts that fine‐scale heterogeneity (e.g., in soil moisture, surface leaf litter) could promote stable coexistence of these species. To the extent that seedlings of encroaching woody species (e.g., pines, shrubs) disproportionately get established in microsites where mesophytic grasses and their litter are absent and more mineral soil is exposed (Brewer, 2002; Landers, 1991), wetland forbs as a group may be vulnerable to competition from pines than are mesophytic species. On the other hand, wetland forbs may be more likely to co‐occur with other wetland forbs than with mesophytic grasses, because they have similar environment requirements and are more‐or‐less equally fit. As a result, their loss with pine encroachment may result from having lower fitness in the presence of pines than do mesophytic grasses.

The objectives of the current study were threefold. First, we described 22 traits in 52 herbaceous species encountered at three wet pine savanna sites and calculated pairwise distances in trait space among all species. Traits included nutrient acquisition adaptations (e.g., carnivory), flowering phenology, fire‐related traits, photosynthetic pathway, specific leaf mass, leaf water content, and size‐related traits, to name a few. Second, we used similarity profile permutation tests and k‐R clustering to identify a maximum number of statistically significant functional groups and then used randomization tests to determine if the loss of functional groups associated with pine encroachment was greater (or less) than expected by chance. Third, we used a new application of permutation‐based multivariate dispersion tests to test whether species that responded to similarly to pine encroachment were more or less likely to co‐occur than expected by chance.

We tested three hypotheses, as outlined in Figures 1 and 2a,b using randomization tests: (a), the absence of functional groups near pines was greater than expected from random species losses (Figure 1); (b) categories of species that responded non‐randomly to pine encroachment (i.e., sensitive or tolerant response categories) contained species that were less likely to co‐occur by chance in areas in savannas away from pines, consistent with niche partitioning (Figure 2a), and (c) sensitive and/or tolerant response categories contained species that were more likely to co‐occur by chance in areas away from pines, consistent with fitness similarity (Figure 2b).

## 2 MATERIALS AND METHODS

### 2.1 Sampling design

Data for the chronosequence study come from measurements of plant species richness and composition in 1997 at three sites in Desoto National Forest in southeastern Mississippi, USA (see also Brewer, 2017). The three sites (hereafter Sandy Creek, Wolf Branch, and Little Red Creek) contained open wet savannas, historically with a sparse canopy of fire‐tolerant *Pinus palustris* (longleaf pine). Poor drainage, low pH, and periodic fires resulted in a groundcover plant community dominated by grasses, sedges, and carnivorous pitcher plants (*Sarracenia alata* Alph. Wood). *Pinus elliottii* (slash pine), which is less tolerant of fire than longleaf pine, invaded most savannas in the region following logging and fire exclusion in the 1900s (Harper, 1914; Heyward, 1939). It was the dominant overstory species at all three sites in 1997. From the early 1980s to 1996, the three sites were burned once every 3 years in the winter, which was effective at halting encroachment by slash pine and other fire‐sensitive tree species (Hinman et al., 2008). Plant species richness is significantly lower near pines and decreases with increasing age of pines, which is positively correlated with size (Brewer, 1998, 2017). Although pine seedling establishment benefits from exposed mineral soil, there is no evidence that seedling establishment disproportionately occurs in patches of low herb diversity (Hinman & Brewer, 2007; Hinman et al., 2008). Hence, patches of groundcover vegetation associated with pines of different ages provide a chronosequence of species loss associated with pine encroachment during the period of fire exclusion.

To quantify differences in plant species richness and composition between open grass‐sedge areas and areas near pines, we sampled 16 plots (0.25 m × 0.25 m) located within a ~0.5‐ha open portion of the savanna at each site. Each plot was located greater than 5 m from the closest slash pine tree. In addition, 16, 16, and 14 plots, respectively, were established within woody thickets adjacent to (i.e., within 1 m) of a slash pine greater than 5 cm diameter‐at‐breast‐height (dbh) at Wolf Branch, Little Red Creek, and Sandy Creek in the general vicinity (within 20 m) of the plots in the open areas. Censuses at all three sites were conducted in May and September of 1997. Results of both censuses were combined into a single response, which included presence/absence of each species encountered during the season in which it was most likely to be encountered and identified (e.g., when flowering). Presence/absence data are provided in Table S1. Prior to sampling in 1997, Wolf Branch was last burned in January of 1996, Sandy Creek was last burned in November of 1996, and Little Red Creek was last burned in January of 1995.

### 2.2 Trait analysis

We described all herbaceous vascular plant species encountered in plots and that we could identify with respect to 22 traits (Table S2). We described both categorical (e.g., carnivory and photosynthetic pathway) and quantitative traits. In addition to traits that were potentially related to niche differences (e.g., seasonal timing of flowering, nutrient‐acquisition strategy), we also measured traits that were more directly related to position in a competitive hierarchy (e.g., size, specific leaf mass), and still others that were indicative of fire‐mediated phenotypic plasticity (fire‐stimulated flowering, fire‐stimulated emergence and vegetatively dormant in years without fire). Some quantitative traits were measured on a continuous scale (e.g., specific leaf mass, leaf moisture and flowering (spore‐producing) season), whereas others (e.g., maximum root depth, rhizome thickness, rhizome length and root porosity) involved classifying species into one of two categories: greater than the median value or less than or equal to the median value of the species pool. For some binary variables, there were inadequate data to classify some species. For example, for the trait “fire‐stimulated emergence”, evidence for or against fire‐stimulated emergence was lacking for some species. Accordingly, species were classified as either exhibiting fire‐stimulated emergence or not known to exhibit fire‐stimulated emergence. To account for the heterogeneous data structure (continuous, binary, missing observations), we calculated a Gower similarity coefficient (Gower, 1971).

### 2.3 Identification of functional groups

We used group‐average hierarchical clustering combined with similarity profile permutation analysis (using Primer version 7 software), to define a maximum number of functional groups exhibiting significant multivariate structure (Clarke et al., 2016). We then refined group number and membership using k‐R cluster analysis, a non‐parametric analog to k‐means cluster analysis (Clarke et al., 2016). Analyses were performed using a Gower similarity matrix derived from the species by trait matrix.

### 2.4 Assessment of absences of functional groups near pines

Once the functional groups were identified, we used a randomization approach analogous to rarefaction to determine if the number of functional groups near pines was lower than expected from a random loss of species (Gotelli & Colwell, 2001). For each of the three sites, we randomly permuted 1,000 functional group matrices, holding the number of species per plot constant, as well as the overall number of occurrences of each species at each site. Hence, only the number of functional groups per plot was allowed to vary with the randomization. We performed the randomization procedure using the *permatswap* function in the vegan package of R (version 2.4, Hardy, 2008). Once we obtained the randomly permuted matrices, we then calculated the difference between the observed number of functional groups (observed functional group richness, *G*_obs_) and the expected functional group richness (*G*_exp_) for each plot, *i*, near pines. We averaged these differences across all plots near pines and divided the average by the standard deviation to obtain a standardized effect size of pine encroachment on functional group richness (*SES*_G_):

(1) $${\text{SES}}_{G}=\frac{{\bar{\left(G_{\text{obs}}‐G_{\exp}\right)}}_{i}}{\text{STDEV}{\left(G_{\text{obs}}‐G_{\exp}\right)}_{i}}$$

In addition to assessing losses of functional groups near pines, we also quantified changes in functional diversity directly from the functional traits, by calculating functional dispersion of samples near and away from pines (using the *dbFD* function in the *FD* package of R). The functional dispersion of a sample of species is a measure of the average distance of species from the centroid in trait space and, unlike the number of functional groups, is independent of the number of species in the sample (Laliberté & Legendre, 2010). We used linear mixed models (*lmerTest* in R) to test for differences in functional dispersion between plots near pines and those away from pines. Site was considered a random effect.

### 2.5 Identification of pine response categories

In addition to calculating differences between observed and expected functional group richness, we used the randomization procedure to determine which functional groups were more or less likely to lose species with encroachment than expected by chance. We calculated observed and expected within‐group species richness for each functional group, *g*, at each plot, *i*, near a tree, took the difference (observed – expected) for each randomized pseudoreplicate, and calculated the mean and standard deviation of these among plots at each site. We divided the mean by the standard deviation to produce a standardized effect size (*SES_within‐group species richness_*) for each functional group.

(2) $${\text{SES}}_{\text{within ‐ group species richness}\left(g\right)}=\frac{{\bar{\left(S_{\text{obs}}‐S_{\exp}\right)}}_{i}}{\text{STDEV}{\left(S_{\text{obs}}‐S_{\exp}\right)}_{i}},$$

where *S* is the number of species within functional group, *g*. Positive values indicated that the observed species richness within a functional group near trees was greater than expected by chance and thus indicated that the functional group was relatively more tolerant of pines. Negative values indicated that the observed species richness within a functional group near trees was lower than expected by chance and thus indicated that the functional group was relatively more sensitive to pines. Some functional groups contained too few species or were too rare to produce 1,000 unique random permutations. Accordingly, the responses of these functional groups should be viewed with caution, as would be the case for any analysis of rare species or groups.

Once we identified functional groups that were more or less likely to lose species than expected by chance, we re‐grouped species in these functional groups into two pine response categories: the tolerant response category, which consisted of those species that were displaced by pine encroachment at a rate that was less than expected by chance, and the sensitive response category, which contained those species that were displaced by pine encroachment at rate that was greater than expected by chance (hereafter, sensitive species).

### 2.6 Patterns of co‐occurrence of sensitive and tolerant species away from pines

To determine whether species within a pine response category were more or less likely to co‐occur than expected by chance in areas away from pines, we pruned the species × plot matrix for each site to include only those species belonging to one of the two pine response categories and only those plots away from pines. We then used the resulting matrix to generate a Bray‐Curtis distance (dissimilarity) matrix using species (rather than plots) as observations. The result provided a matrix of distances among species with respect to their occurrence in plots away from pines for each site. The matrix was then subjected to a multivariate dispersion test (using *betadisper* in R), wherein we calculated the distance of each species from its category centroid (i.e., either the tolerant category centroid or the sensitive category centroid). We then calculated the mean and standard deviation of distances separately for tolerant and sensitive species to generate a standardized average distance (dispersion) for each site (*Disp*_(tolerant)_ and *Disp*_(sensitive)_). We compared the observed average distance for each response category to that expected by chance by randomizing occurrences of each species 1,000 times using *permatswap* and then calculating the average distance of each species to its response category centroid using *betadisper*. For each response category, an observed average distance that was greater than expected by chance indicated that species within a response category (e.g., sensitive species) were less likely to co‐occur in areas away from pines than expected by chance. Such a response would support the hypothesis that species in the same response category are not likely to co‐occur, perhaps because they occupy similar niches (Figure 2a). In contrast, an observed average distance that was less than expected by chance indicated that species within a response category were more likely to co‐occur than expected by chance. Such a response would support the hypothesis that species in the same response category are likely to co‐occur perhaps because of similar fitness (competitive abilities) and/or abiotic requirements (Figure 2b).

## 3 RESULTS

### 3.1 Functional group responses to pine encroachment

Using 22 traits in 52 herbaceous species, similarity profile permutation tests and k‐R clustering identified a maximum of 17 statistically significant functional groups (Table S3). Functional group membership to some extent was related to phylogeny. For example, all fall‐flowering grasses formed a single functional group, as did the two species of *Sarracenia*, the two species of *Polygala* L., the two species of club moss, and the two species of orchids. Other groups contained a mix of species that shared ecologically important traits (e.g., perennials with thick rhizomes or corms and high leaf moisture: *Chaptalia tomentosa* Vent., both *Eriocaulon* L. species, *Hypoxis wrightii* (Baker) Brackett, *Xyris drummondii* Malme, and *Zigadenus glaberrimus* Michx.; Table S3).

**FIGURE 3:**
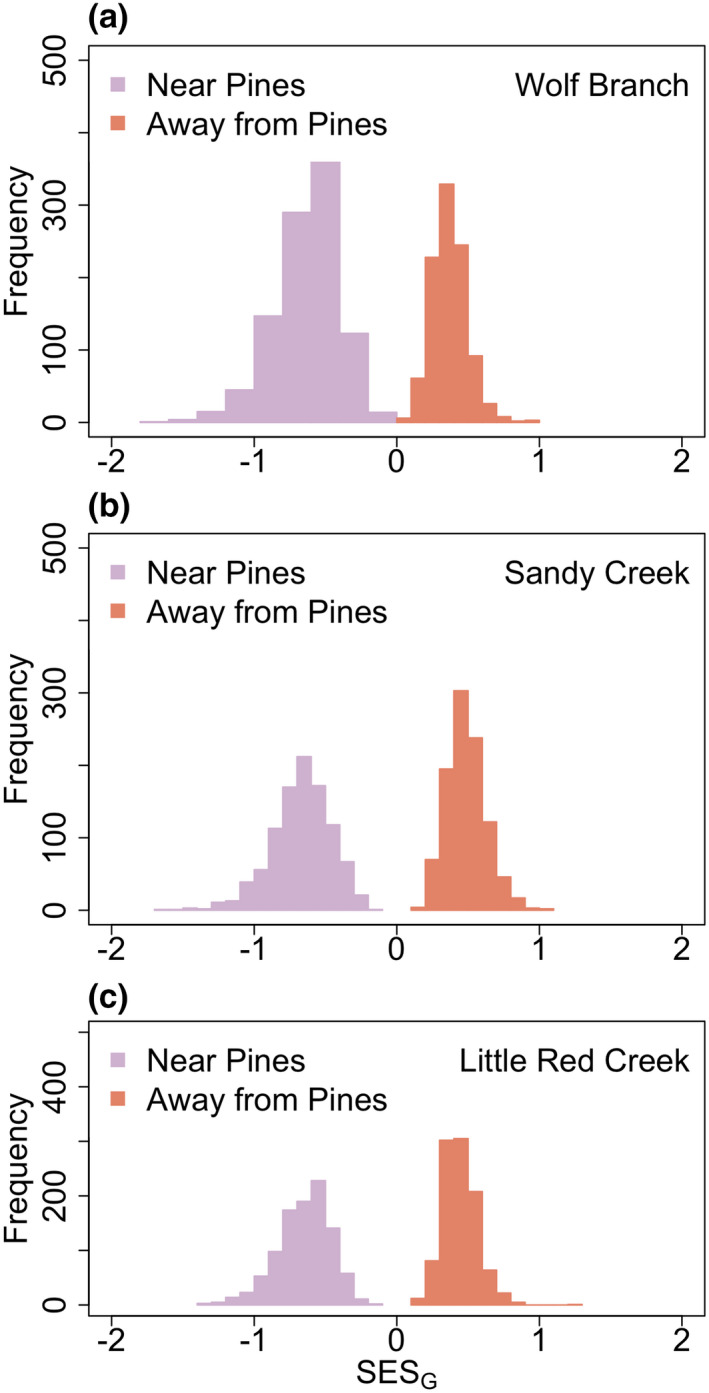
Distribution of effects of pine encroachment on functional group richness. In all panels, purple histograms represent plots near pines, and orange histograms represent plots in the open, away from pines. Histograms are based on 1,000 random permutations of functional group matrices. (a) Wolf Branch, (b) Sandy Creek and, (c) Little Red Creek

**FIGURE 4:**
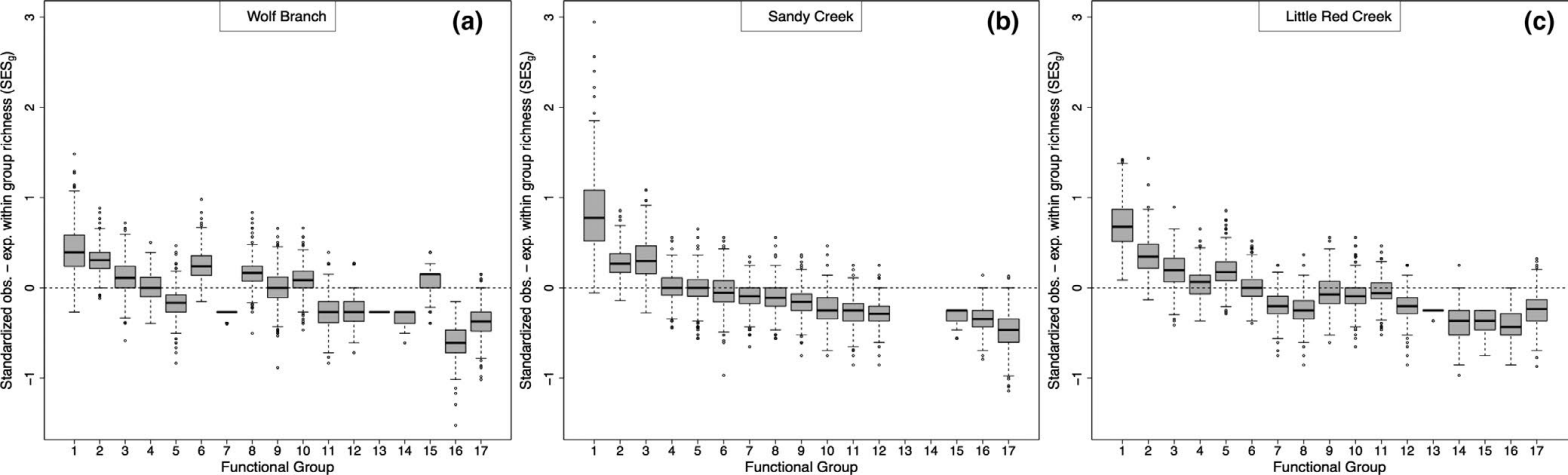
Boxplots of observed species richness within‐functional group minus within‐group species richness expected from random species losses near pines at three sites: (a) Wolf Branch, (b) Sandy Creek, and (c) Little Red Creek. Average differences in observed and expected richness among plots at each site were divided by the standard deviation to obtain a standardized effect size of pines on within‐group species richness. Distributions for 17 functional groups are based on 1,000 runs for each site. Positive values indicate that observed species richness for that functional group near pines was greater than expected from random species loss, whereas negative values indicate the opposite

The reduction in the number of functional groups from pine encroachment was greater than expected from random species losses at all three sites (Figure 3). Mean *SES*_G_ (± standard error) was −0.634 (0.0002), −0.673 (0.0002), and −0.652 (0.0002) for Wolf Branch, Sandy Creek, and Little Red Creek, respectively (randomization *p* < .001). Functional groups that lost species near trees at rates greater than expected by chance at least one site included wetland perennials with thick rhizomes or corms and high leaf moisture (FG 17; at Sandy Creek and Wolf Branch), spring‐flowering perennials of medium height and shallow roots; that is, *Drosera tracyi*, *Erigeron vernus*, and *Rhexia lutea* (FG 16) at all three sites, *Polygala* spp. (FG 15) at Sandy Creek and Little Red Creek, orchids (FG 13; at Wolf Branch and Little Red Creek), the semi‐woody *Hypericum brachyphyllum* (Spach) Steud. (FG 12) at Sandy Creek and Wolf Branch, a short‐lived non‐carnivorous plant that is not a *Polygala* (*Scleria reticularis* Michx.; FG 14) at Wolf Branch and Little Red Creek, and *Sarracenia* spp. (FG11) at Wolf Branch and Sandy Creek (Figure 4). Interpretations of losses of rare functional groups or species at a site (e.g., FGs 14 and 15) should be viewed with caution, given the inadequate number of unique permutations.

Functional groups that were less likely to be lost than expected by chance included the mostly short C_3_ grasses and sedges that produced seed banks (FG 1; e.g., *Dichanthelium* (Hitchc. & Chase) Gould spp., *Rhynchospora* Vahl spp.) at all three sites, the tall fall‐flowering grasses (FG 2; e.g., Andropogonae, *Muhlenbergia expansa*, *Anthaenantia rufa* (Nutt.) Schult., *Aristida palustris* (Chapm.) Vasey) at all three sites, and medium‐height wetland dicots and non‐graminoid monocots that exhibited fire‐stimulated flowering (FG 3; *Bigelowia nudata* Michx. DC, *Coreopsis linifolia* Nutt., *Lachnanthes caroliniana* (Lam.) Dandy, *Lophiola aurea* Ker Gawl., *Triantha racemosa* (Walter) Small, and *Xyris ambigua* Bey. ex Kunth. at Sandy Creek; Figure 4).

**FIGURE 5:**
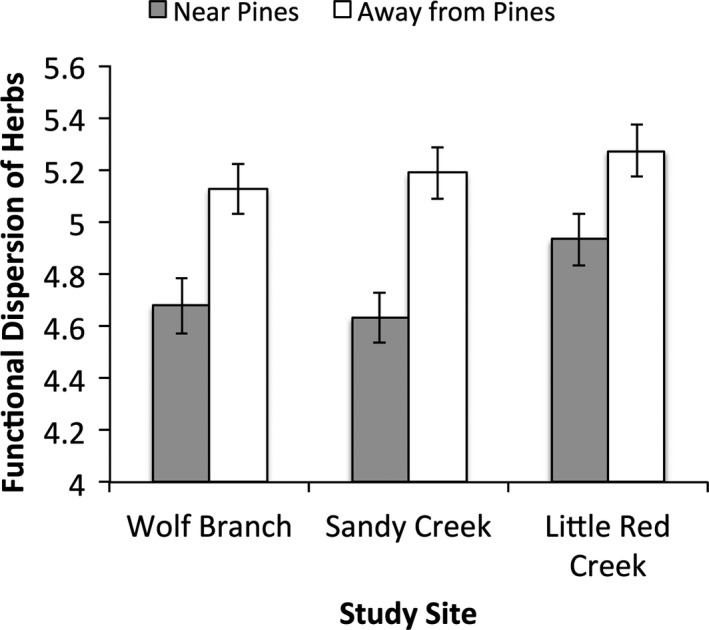
Functional dispersion near and away from pines at Wolf Branch, Sandy Creek, and Little Red Creek. Values are mean Euclidean distance to the community centroid in trait space in principal coordinates analysis axis score units ±1 standard error derived from the mean squared error

As with the reduction in the number of functional groups, functional dispersion was significantly lower near pines than away from pines at all three sites (*F*_tree proximity_ = 30.21; *p* ≪ 0.01; Satterthwaite approximate *df* = 1, 90.08; Figure 5). A linear fixed‐effects model showed that the site × tree proximity interaction was not statistically significant (*F*_2,88_ = 0.59; *p* = .56).

### 3.2 Patterns of co‐occurrence of species sensitive to or tolerant of pine encroachment

**FIGURE 6:**
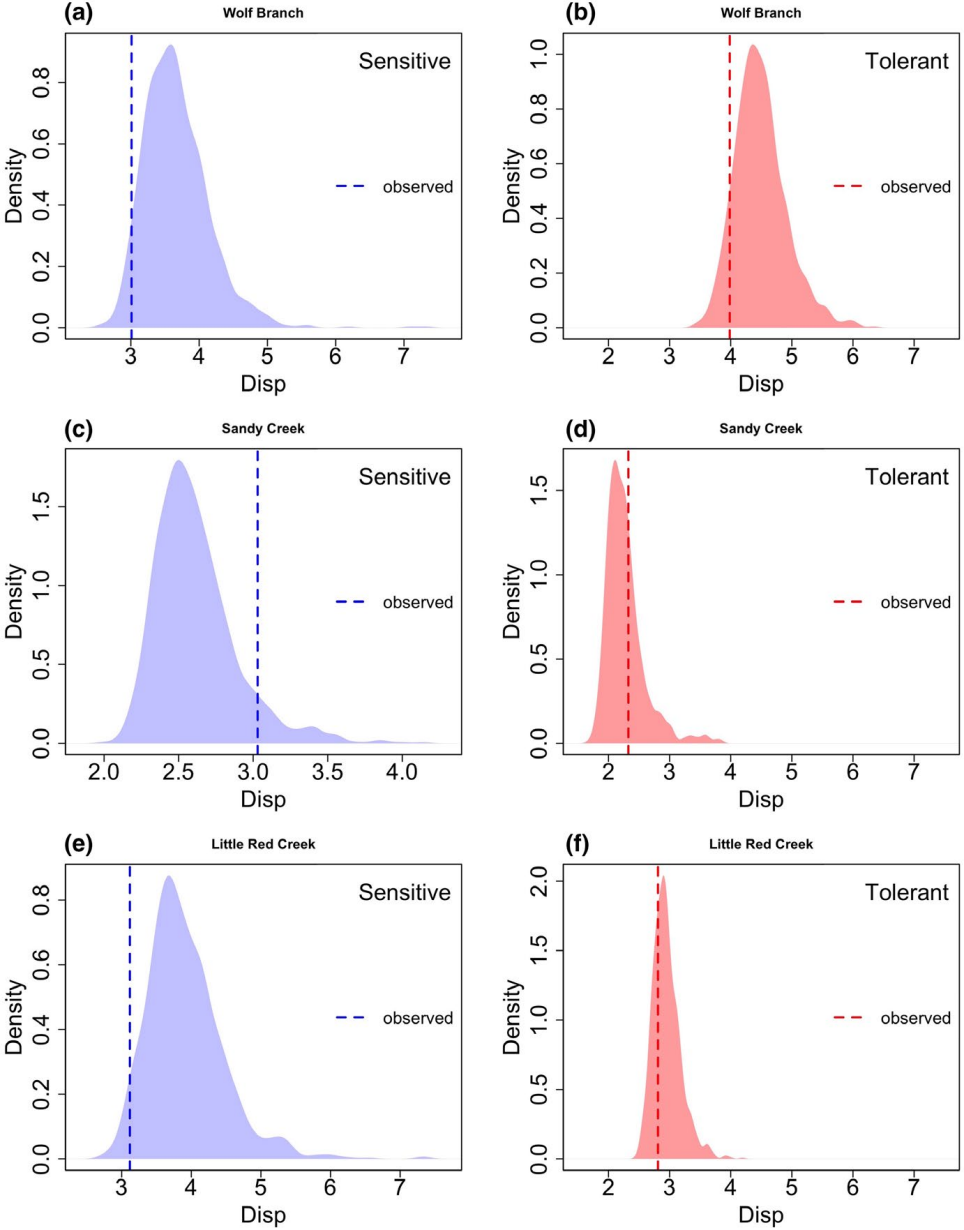
Density plots of the average distance of sensitive (a, c, e) and tolerant (b, d, f) species in sampling plot space to each response category's location centroid (*Disp*). *Disp*_obs_ is represented by dashed vertical lines for each of three sites: (a, b) Wolf Creek, (c, d) Sandy Creek, and (e, f) Little Red Creek. Randomized distributions of dispersion expected by chance (*Disp*_exp_) were obtained by randomizing occurrences of each species 1,000 times using *permatswap* in R and then calculating the average distance of each species to its response category centroid using *betadisper*. For each response category, an observed average distance that was less than expected by chance indicated that species within a response category (e.g., sensitive species at Wolf Branch) were more likely to co‐occur in areas away from pines than expected by chance

Considering only those species within functional groups that were lost with pine encroachment at a rate not expected by chance, patterns of co‐occurrence were not consistent with a hypothesis of niche partitioning. Species within the sensitive competitive response group generally were not less likely to co‐occur with one another in areas away from pines (Figure 6). In fact, species within the sensitive response group were more likely to co‐occur away from pines at Wolf Branch (Wolf Branch, randomization *p* = .044; i.e., 956 of 1,000 *Disp*_(exp)_ were greater than the *Disp*_(obs)_ Figure 6a) and showed a similar trend at Little Red Creek (*p* = .033; Figure 6e). At Sandy Creek, there was a weak trend suggestive of sensitive species co‐occurring less often than expected by chance, but the result was not statistically significant at the 0.05 level (randomization *p* = .08; Figure 6c). Patterns of co‐occurrences of species within tolerant functional groups varied among the three sites. At Wolf Branch, tolerant species showed a weak trend of co‐occurring in open areas more often than expected by chance (randomization *p* = .097; Figure 6b). At Sandy Creek, tolerant species were neither more nor less likely to co‐occur in open areas than expected by chance (randomization *p* = .33; Figure 6d). At Little Red Creek, tolerant species were not significantly more or less likely to co‐occur than expected by chance (randomization *p* for *Disp*_(exp)_ < *Disp*_(obs)_ = 0.283 and 0.717 for *Disp*_(exp)_ > *Disp*_(obs)_; Figure 6f).

## 4 DISCUSSION

### 4.1 Functional herb diversity was lower near pines and dominated by graminoids

In the current study, we found that the disassembly of herb assemblages following encroachment by pines was not random at any of the three sites examined. While most functional groups were more likely to be absent near pines than expected by chance, functional groups that were less likely to be absent near pines than expected by chance included the tall fall‐flowering grasses and the mostly short C_3_ grasses and sedges that produced seed banks. In addition, medium‐height wetland forbs and non‐graminoid monocots that exhibited fire‐stimulated flowering were also less likely to be absent near pines than expected by chance. Functional groups that were more likely to be absent near pines than expected by chance at one or more sites included spring‐flowering perennials with thick rhizomes or corms and high leaf moisture, wetland perennials with shallow roots, *Polygala* spp., pitcher plants (i.e., *Sarracenia* spp. at Wolf Branch), orchids, the semi‐woody *Hypericum brachyphyllum*, the short‐lived sedge, *Scleria reticularis*, and club mosses. Responses of the latter five groups must be interpreted with caution, given that they consisted of only 1 or 2 species.

The elevated and non‐random loss of functional groups near pines contrasts somewhat with what was previously reported in this system with regard to individual species losses (Brewer, 2017). In the previous study, losses of species near pines were not different than expected by chance. Differences in the results of the two studies may be related to the fact that quantification of species losses in the previous study was based on presence–absence data of species, whereas the functional group analysis here was based on “abundance”‐weighted functional groups, wherein the “abundance” of a functional group in a given plot was quantified by counting the number of species in that functional group in that plot (analogous to weighting each species by the number of individuals of that species). Analyses based on abundance‐weighted data may be more sensitive to deterministic processes than are analyses based on presence‐absence data (Tucker et al., 2016).

The finding that functional group richness near trees was lower than expected from random species losses at all three sites was corroborated by functional trait dispersion being lower near pines than away from pines. A limitation of examining functional diversity losses from reduction of functional group richness is that losses of functional groups depend in part on how species are assigned to functional groups. Others have argued that the approach of assigning species to functional groups a posteriori based on measured traits is more objective than assigning species to functional groups a priori (Laliberté & Legendre, 2010). We agree with this assertion, but acknowledge that a shortcoming of the a posteriori approach we used is that generalists must be assigned to a particular functional group, which may not be warranted. Quantifying functional diversity by calculating functional dispersion (the mean distance to a community centroid in trait space) does not require the creation of functional groups and thus sidesteps the problem of assigning generalists to one or another functional group. A limitation of the functional dispersion approach, however, is that it does not provide a straightforward way of identifying which species are most vulnerable to loss. We assert that the combination of examining functional group losses, losses of species within groups, and reductions of functional dispersion irrespective of species identity addresses the inherent trade‐offs of these different approaches.

### 4.2 Species sensitive to pine encroachment were more likely to co‐occur at two of three sites

Contrary to the predictions of niche partitioning, species within the response category that was sensitive to pine encroachment were more, not less, likely to co‐occur in small plots in non‐encroached areas than expected by chance at two of the three sites. Hence, species coexistence and the maintenance of fire‐scale species diversity in the hyper‐diverse savannas studied here appear to be driven more by fitness similarities than by niche differences among herbs. These results cast doubt on the importance of niches in reducing competition a local scale, as defined by the response categories identified here. Rather, they are consistent with the hypothesis that there was a competitive hierarchy, in which the species within the sensitive response category had more‐or‐less equally low fitness (Figure 2b). This conclusion is supported by the observation that the most species‐rich plots away from pines contained greater numbers of species within the sensitive response group rather than a more equitable mix of species from sensitive and tolerant competitive response groups (Brewer, 1998). These findings are consistent with those of previous studies examining the relative importance of niche differences and fitness similarity in promoting species coexistence in other herbaceous communities (Bennett et al., 2016; Carmona et al., 2019; Götzenberger et al., 2012).

### 4.3 Caveats

Although we found no evidence that species with similar functional niches were less likely to co‐exist than were species with different functional niches, these results do not mean that density‐dependent stabilizing mechanisms were absent in this system (Chesson, 1991). Negative feedbacks associated with host‐specific natural enemies could promote species coexistence among herbaceous species in the absence of woody encroachment (Bever, 1994; Connell, 1971; Janzen, 1970; Reynolds et al., 2003). In such a case, the morphology‐based functional group to which a species belonged would not be a good proxy for its niche. In addition, the chronosequence used here is not a perfect substitute for long‐term experiments and thus caution is warranted when interpreting the results. We cannot conclude with certainty that the absence of any given species from a plot near a pine was due to displacement by that pine. It is of course possible that the species was never there in the first place. Another possibility is that its mortality near pines was no greater near than away from pines, but recruitment, either from a seed bank or from dispersal, was lower near pines. Long‐term monitoring of populations of herbaceous species before and after establishment by pines is necessary to fully understand the processes underlying the patterns observed in this study. These limitations notwithstanding, we maintain that the identities of the species that were absent near pines were remarkably consistent among plots and among sites in this system. Although small‐scale alpha‐diversity is relatively high in plots located away from pines, beta‐diversity (i.e., variation in species composition among plots away from pines) is relatively low in the wet pine savannas studied here (Brewer, 2017).

Two other potential limitations of the current study relate to the sampling procedure. First, although the analysis of losses of functional groups accounted for the number of species within each group, the analysis of patterns of species occurrence away from pines did not account for the number of individuals within each species. Hence, the analysis of species composition patterns away from pines may have lacked sufficient power to detect niche differences. However, the approach was sufficiently powerful to detect patterns of co‐occurrence of species within response categories that were greater than expected by chance at two of the three sites. Another perhaps more serious limitation is that we underestimated plant species diversity and thus potential niche differences, especially at Wolf Branch and Little Red Creek. Although all three sites were burned on average once every 3 years from 1980 to 1996, only one of the three sites, Sandy Creek, was burned within 1 year of the censusing in 1997. It has been well established that fire frequency and time since the most recent fire affect detectable plant species diversity in pine savannas (Glitzenstein et al., 2003; Hinman & Brewer, 2007; Palmquist et al., 2014). The effect is so dramatic in some cases that even a reduction from annual burning to burning once every two or more years or waiting until the second year after a fire can result in a significant reduction in detectable plant species diversity (Glitzenstein et al., ,,^2003^, ^2012^; Hinman & Brewer, 2007; Palmquist et al., 2014). Accordingly, had Wolf Branch and Little Red Creek been censused the first year after a fire, we might have detected a greater number of species and thus niche differences. Indeed, at Sandy Creek was there a trend (albeit not significant, *p* = .112) toward sensitive species co‐occurring less often than expected by chance (Figure 4). This finding raises the intriguing possibility of whether frequent fires (often viewed as an alternative to niche differences as an explanation for high species diversity) (Wilson et al., 2012) are necessary to detect niche differences among co‐occurring species within fire‐dependent savannas (Brewer, 2006).

## CONFLICT OF INTEREST

None declared.

## AUTHOR CONTRIBUTIONS

**Stephen Brewer:** Conceptualization (lead); data curation (lead); formal analysis (supporting); funding acquisition (lead); investigation (lead); methodology (equal); project administration (lead); resources (lead); software (equal); supervision (lead); validation (equal); visualization (equal); writing–original draft (lead); writing–review and editing (equal). **Peter Zee:** Conceptualization (supporting); formal analysis (lead); methodology (equal); software (equal); validation (equal); visualization (equal); writing–review and editing (equal).

## Supporting information

Table S1

Table S2

Table S3

## Data Availability

All data used in the analyses are provided in the three supplemental tables associated with this manuscript. Table S1 contains the species presence/absence data for all plots sampled data. Table S2 contains the trait data used to define functional groups. Table S3 contains the functional groups data. All are available through the Dryad Digital Repository: Table S1 ‐ https://doi.org/10.5061/dryad.1vhhmgqs7; Table S2 ‐ https://doi.org/10.5061/dryad.qjq2bvqfq; Table S3 ‐ https://doi.org/10.5061/dryad.t1g1jwt1z.
