# Supplementary material for: Functional diversity and coexistence of herbaceous plants in wet, species‐rich savannas
Source: Ecol Evol. 2021 Mar 22;11(10):5111–20. doi: 10.1002/ece3.7404 (PMC8131808; doi:10.1002/ece3.7404)
Supplement: Supplementary file 2 — Table S2 [file ECE3-11-5111-s002.pdf]

Table S2: Trait Data Used to Generate Functional Groups. **Brewer JS, Zee P.** Functional diversity and coexistence of herbaceeous plants in wet, species-rich savannas. Ecology and Evolution.

Species	Trait 1	Trait 2	Trait 3	Trait 4	Trait 5	Trait 6	Trait 7
Andropogon mohrii	1	0	1	0	1	1	0
Andropogon virginicus	1	0	1	0	1	1	0
Anthaenantia rufa	1	0	1	0	1	1	0
Aristida palustris	0.96	0	1	0	1	1	0
Balduina uniflora	0.84	1	0.5	1	1	0	0
Bigelowia nudata	0.96	1	0.5	1	1	1	0
Calopogon pallidus	0.39	1	0.5	1	1	1	0
Carphephorus pseudoliatris	0.88	1	0.5	1	1	1	0
Chaptalia tomentosa	0.1	1	0	1	1	1	0
Coreopsis linifolia	0.96	1	0.5	1	1	1	0
Ctenium aromaticum	0.63	0	1	0	1	1	0
Dichanthelium ensifolium	0.67	0	0	1	1	1	0
Dichanthelium lucidum	0.67	0	0	1	1	1	0
Drosera capillaris	0.51	1	0	1	1	0	1
Drosera tracyi	0.38	1	0.5	1	1	0	1
Erigeron vernus	0.15	1	0.5	1	1	0	0
Eriocaulon compressum	0.78	1	0.5	1	1	1	0
Eriocaulon decangulare	0.78	1	0.5	1	1	1	0
Helianthus heterophyllus	0.96	1	1	1	1	1	0
Hypericum brachyphyllum	0.63	0	0.5	1	1	0	0
Hypoxis wrightii	0.08	1	0	1	1	1	0
Lachnocaulon anceps	0.67	1	0	1	1	1	0
Lacnanthes caroliniana	0.72	1	0.5	1	1	1	0
Linum medium/floridanum	0.78	0	0.5	1	1	1	0
Lophiola aurea	0.27	1	0.5	1	1	1	0
Lycopodiella alopecuroides	0.84	0	0.5	1	0	0	0
Pseudolycopodiella caroliniana	0.84	0	0.5	1	0	0	0
Muhlenbergia expansa	1	0	1	0	1	1	0
Pinguicula planifolia	0.08	1	0	1	1	1	1
Pogonia ophioglossoides	0.08	1	0.5	1	1	1	0

Species	Trait 1	Trait 2	Trait 3	Trait 4	Trait 5	Trait 6	Trait 7
Polygala chapmanii	0.63	0	0	1	1	0	0
Polygala cruciata	0.78	0	0	1	1	0	0
Rhexia alifanus	0.61	0	1	1	1	1	0
Rhexia lutea	0.3	0	0.5	1	1	0	0
Rhynchospora baldwinii	0.75	0	0.5	1	1	1	0
Rhynchospora chapmanii	0.84	0	0.5	1	1	1	0
Rhynchospora latifolia	0.61	1	0.5	1	1	1	0
Rhynchospora oligantha	0.75	0	0	1	1	1	0
Rhynchosporaplumosa	0.75	0	0.5	1	1	1	0
Sarracenia alata	0.03	1	0	1	1	1	1
Sarracenia psittacina	0.03	1	0.5	1	1	1	1
Schizachyrium tenerum	0.96	0	1	0	1	1	0
Scleria pauciflora	0.72	0	0.5	1	1	1	0
Scleria reticularis	0.72	0	0.5	1	1	1	0
Tiedemannia filiformis	0.75	1	1	1	1	0	0
Triantha racemosa	0.63	1	0.5	1	1	1	0
Utricularia subulata	0.25	1	0	1	1	0	1
Xyris ambigua	0.51	1	0.5	1	1	1	0
Xyris baldwinii	0.51	1	0	1	1	1	0
Xyris caroliniana	0.51	1	0.5	1	1	1	0
Xyris drummondii	0.51	1	0	1	1	1	0
Zigadenus glaberrimus	0.72	1	0.5	1	1	1	0

Species	Trait 8	Trait 9	Trait 10	Trait 11	Trait 12	Trait 13	Trait 14
Andropogon mohrii	0	1	1	0.007	0.66	0	0
Andropogon virginicus	0	1	1	0.00761	0.63	0	0
Anthaenantia rufa	0	1	1	0.006	0.69	0	0
Aristida palustris	0	1	1	0.006	0.69	0	0
Balduina uniflora	0	1	1	0.0045	0.74	0	0
Bigelowia nudata	0	1	1	0.00417	0.81	0	0
Calopogon pallidus	0	1	1	0.0037	0.77	0	1
Carphephorus pseudoliatris	0	1	1	0.01128	0.68	0	1
Chaptalia tomentosa	0	1	1	0.00489	0.79	0	1
Coreopsis linifolia	0	1	1	0.00424	0.93	1	0
Ctenium aromaticum	0	1	1	0.0109	0.55	0	1
Dichanthelium ensifolium	0	1	1	0.0075	0.75	0	0
Dichanthelium lucidum	0	1	1	0.008	0.63	0	0
Drosera capillaris	0	0	1	0.00735	0.5	0	0
Drosera tracyi	0	1	1	0.00426	0.8	0	0
Erigeron vernus	0	1	1	0.004	0.76	0	0
Eriocaulon compressum	0	1	1	0.00329	0.84	0	1
Eriocaulon decangulare	0	1	1	0.003	0.82	0	1
Helianthus heterophyllus	0	1	1	0.009	0.6	0	0
Hypericum brachyphyllum	1	1	1	0.00955	0.48	0	0
Hypoxis wrightii	0	1	1	0.00342	0.63	0	1
Lachnocaulon anceps	0	1	1	0.0026	0.87	0	0
Lacnanthes caroliniana	0	1	1	0.0036	0.86	1	0
Linum medium/floridanum	0	1	1	0.00375	0.67	0	0
Lophiola aurea	0	1	1	0.00413	0.74	1	0
Lycopodiella alopecuroides	0	1	1	0.00184	0.82	0	0
Pseudolycopodiella caroliniana	0	1	1	0.002	0.82	0	0
Muhlenbergia expansa	0	1	1	0.01049	0.42	0	0
Pinguicula planifolia	0	1	1	0.003	0.79	0	0
Pogonia ophioglossoides	0	1	1	0.0034	0.78	0	1
Polygala chapmanii	0	0	1	0.003	0.82	0	0
Polygala cruciata	0	0	1	0.0025	0.9	0	0
Rhexia alifanus	0	1	1	0.00182	0.79	0	1
Rhexia lutea	0	1	1	0.00198	0.77	0	0
Rhynchospora baldwinii	0	1	1	0.003	0.79	0	0
Rhynchospora chapmanii	0	1	1	0.0048	0.67	0	0
Rhynchospora latifolia	0	1	1	0.00362	0.75	1	0
Rhynchospora oligantha	0	1	1	0.0048	0.67	0	0
Rhynchosporaplumosa	0	1	1	0.0048	0.66	0	0
Sarracenia alata	0	1	1	0.00681	0.65	0	1

Species	Trait 8	Trait 9	Trait 10	Trait 11	Trait 12	Trait 13	Trait 14
Sarracenia psittacina	0	1	1	0.006	0.64	0	1
Schizachyrium tenerum	0	1	1	0.0071	0.66	0	0
Scleria pauciflora	0	1	1	0.005	0.73	0	0
Scleria reticularis	0	0	1	0.00233	0.71	0	0
Tiedemannia filiformis	0	1	1	0.0054	0.86	0	0
Triantha racemosa	0	1	1	0.006	0.69	0	0
Utricularia subulata	0	0	0	0.001	0.9	0	0
Xyris ambigua	0	1	1	0.00611	0.78	0	0
Xyris baldwinii	0	1	1	0.00518	0.71	0	0
Xyris caroliniana	0	1	1	0.00611	0.65	0	1
Xyris drummondii	0	1	1	0.0055	0.71	0	1
Zigadenus glaberrimus	0	1	1	0.00335	0.81	0	1

Species	Trait 15	Trait 16	Trait 17	Trait 18	Trait 19	Trait 20	Trait 21
Andropogon mohrii	0	1	0	0	0	1	1
Andropogon virginicus	0	1	1	0	0	1	0
Anthaenantia rufa	0	1		0	0	1	0
Aristida palustris	0	1		0	0	1	1
Balduina uniflora	0	0	0	0		1	0
Bigelowia nudata	0	0		0		1	1
Calopogon pallidus	1	0	0	1	1	0	0
Carphephorus pseudoliatris	0	0	0	0		1	1
Chaptalia tomentosa	0	0		0	0	1	1
Coreopsis linifolia	0	0	0	0		1	1
Ctenium aromaticum	0	0	0	0	0	1	0
Dichanthelium ensifolium	0	0	1	0		0	0
Dichanthelium lucidum	0	0	1	0		0	0
Drosera capillaris	0	0	1	0	1	0	0
Drosera tracyi	0	0		0		0	0
Erigeron vernus	0	0		0		0	1
Eriocaulon compressum	0	0	1	0		1	1
Eriocaulon decangulare	0	0	1	0		1	1
Helianthus heterophyllus	0	0		0		1	1
Hypericum brachyphyllum	0	0		0	0	0	0
Hypoxis wrightii	0	0		0	0	0	1
Lachnocaulon anceps	0	0		0	0	0	0
Lacnanthes caroliniana	0	0	1	0	0	0	1
Linum medium/floridanum	0	0		0		0	0
Lophiola aurea	0	0	1	0	0	1	1
Lycopodiella alopecuroides	0	0	1	0		0	1
Pseudolycopodiella caroliniana	0	0	1	0		0	0
Muhlenbergia expansa	0	1	0	0	0	1	0
Pinguicula planifolia	0	0		0	0	0	0
Pogonia ophioglossoides	1	0	0	1	1	0	0
Polygala chapmanii	0	1	1	0	1	0	0
Polygala cruciata	0	1	1	0	1	0	0
Rhexia alifanus	0	0	0	0	0	1	0
Rhexia lutea	0	0	0	0	0	0	0
Rhynchospora baldwinii	0	0	1	0		0	0
Rhynchospora chapmanii	0	0	1	0		0	0
Rhynchospora latifolia	0	0	1	0		0	0
Rhynchospora oligantha	0	1	1	0		0	1
Rhynchosporaplumosa	0	1	1	0		0	1
Sarracenia alata	0	0	0	0	1	0	0

Species	Trait 15	Trait 16	Trait 17	Trait 18	Trait 19	Trait 20	Trait 21
Sarracenia psittacina	0	0	0	0	1	0	0
Schizachyrium tenerum	0	1	0	0	0	1	1
Scleria pauciflora	0	1	1	0		0	0
Scleria reticularis	0	0	1	1	1	0	0
Tiedemannia filiformis	0	0		0		1	1
Triantha racemosa	0	0		0	0	1	1
Utricularia subulata	0	0	1	0	1	0	0
Xyris ambigua	0	0	1	0		1	1
Xyris baldwinii	0	0	1	0		1	0
Xyris caroliniana	0	0	1	0		1	0
Xyris drummondii	0	0	1	0		1	0
Zigadenus glaberrimus	0	0	0	0		1	0

Species	Trait 22
Andropogon mohrii	1
Andropogon virginicus	1
Anthaenantia rufa	1
Aristida palustris	
Balduina uniflora	1
Bigelowia nudata	1
Calopogon pallidus	1
Carphephorus pseudoliatris	1
Chaptalia tomentosa	0
Coreopsis linifolia	1
Ctenium aromaticum	1
Dichanthelium ensifolium	0
Dichanthelium lucidum	0
Drosera capillaris	0
Drosera tracyi	0
Erigeron vernus	0
Eriocaulon compressum	0
Eriocaulon decangulare	0
Helianthus heterophyllus	0
Hypericum brachyphyllum	0
Hypoxis wrightii	0
Lachnocaulon anceps	0
Lacnanthes caroliniana	1
Linum medium/floridanum	0
Lophiola aurea	1
Lycopodiella alopecuroides	0
Pseudolycopodiella caroliniana	0
Muhlenbergia expansa	1
Pinguicula planifolia	0
Pogonia ophioglossoides	0
Polygala chapmanii	0
Polygala cruciata	0
Rhexia alifanus	0
Rhexia lutea	0
Rhynchospora baldwinii	0
Rhynchospora chapmanii	0
Rhynchospora latifolia	0
Rhynchospora oligantha	0
Rhynchosporaplumosa	0
Sarracenia alata	0

Species	Trait 22
Sarracenia psittacina	0
Schizachyrium tenerum	1
Scleria pauciflora	0
Scleria reticularis	0
Tiedemannia filiformis	0
Triantha racemosa	1
Utricularia subulata	0
Xyris ambigua	1
Xyris baldwinii	1
Xyris caroliniana	1
Xyris drummondii	0
Zigadenus glaberrimus	0

Trait	
Trait 1	Lateness of midpoint of flowering season (0=March, 1=December)
Trait 2	Scape (leafless flowering stalk) present
Trait 3	Height Category (0 ,0.5, 1 for shortest 1/3, middle 1/3, tallest 1/3)
Trait 4	C3 photosynthetic pathway
Trait 5	Not Prostrate
Trait 6	Presence of Rhizome or corm
Trait 7	Carnivorous
Trait 8	Semi woody
Trait 9	Perennial
Trait 10	Roots present
Trait 11	Specific leaf mass (g/cm2)
Trait 12	Water content of fresh leaves (proportion)
Trait 13	Longer than median rhizome
Trait 14	Thicker (max diam.) than median rhizome or corm
Trait 15	Exhibits vegetative dormancy in years without fire
Trait 16	Leaves predominantly green only after fire
Trait 17	Seed or spore bank likely present
Trait 18	No basal or cauline leaves when flowering
Trait 19	Shows fire-stimulated emergence
Trait 20	Length of deepest root greater than median
Trait 21	Root porosity (aerenchyma) greater median
Trait 22	Shows fire-stimulated flowering

Trait	Response Type
Trait 1	Continuous
Trait 2	Categorical
Trait 3	Ordinal/Rank
Trait 4	Categorical
Trait 5	Categorical
Trait 6	Categorical
Trait 7	Categorical
Trait 8	Categorical
Trait 9	Categorical
Trait 10	Categorical
Trait 11	Continuous
Trait 12	Continuous
Trait 13	Ordinal/Rank
Trait 14	Ordinal/Rank
Trait 15	Categorical
Trait 16	Categorical
Trait 17	Categorical
Trait 18	Categorical
Trait 19	Categorical
Trait 20	Ordinal/Rank
Trait 21	Ordinal/Rank
Trait 22	Categorical

Trait	Source/Method
Trait 1	Published accounts from regional flora manuals
Trait 2	Published accounts from regional flora manuals
Trait 3	Published accounts from regional flora manuals
Trait 4	Published accounts from regional flora manuals
Trait 5	Published accounts from regional flora manuals
Trait 6	Published accounts from regional flora manuals
Trait 7	Published accounts from regional flora manuals
Trait 8	Published accounts from regional flora manuals
Trait 9	Published accounts from regional flora manuals
Trait 10	Published accounts from regional flora manuals
Trait 11	Direct measurement by JS Brewer on 3 to 5 typical leaves
Trait 12	Direct measurement by JS Brewer on 3 to 5 typical leaves
Trait 13	Direct measurement by JS Brewer on 3 to 5 typical rhizomes
Trait 14	Direct measurement by JS Brewer on 3 to 5 typical rhizomes
Trait 15	Field observations and unpublished data from
Trait 16	Field observations and unpublished data from
Trait 17	Published data[1,2] and/or field observations by
Trait 18	Published data and/or field observations by JSB
Trait 19	Published data[2,3,4] and/or field observations by
Trait 20	Published data from Brewer et al. 2011
Trait 21	Published data from Brewer et al. 2012
Trait 22	Published data from Hinman and Brewer 2007

1 - Cohen S, Braham R, Sanchez F. 2004. Seed bankviability in disturbed longleaf pine sites. Restoration Ecology 12: 503–515.

2 - Maliakal SK, Menges ES, Denslow JS. 2000. Community composition and regeneration of Lake Wales Ridge wiregrass flatwoods in relation to time-since-fire. The Journal of the Torrey Botanical

3 - Brewer JS. 1999. Effects of fire, competition, and soil disturbances on regeneration of a carnivorous plant (Drosera capillaris). American Midland Naturalist 141: 28–42.

4 - Brewer JS. 1999. Effects of competition, litter, and disturbance on an annual carnivorous plant (Utricularia juncea). Plant Ecology 140: 159–165.
