# Supplementary material for: Functional diversity and coexistence of herbaceous plants in wet, species‐rich savannas
Source: Ecol Evol. 2021 Mar 22;11(10):5111–20. doi: 10.1002/ece3.7404 (PMC8131808; doi:10.1002/ece3.7404)
Supplement: Supplementary file 3 — Table S3 [file ECE3-11-5111-s003.pdf]

Table S3: Functional Groups and Pine Response Categories. **Brewer JS, Zee P.** Functional diversity and coexistence of herbaceeous plants in wet, species-rich savannas. Ecology and Evolution

Functional Group Defined by k-R (	Functional Group Number	Species	Competitive Response Category
	1	*Dichanthelium ensifolium*	Tolerant
	1	*Dichanthelium lucidum*	Tolerant
	1	*Linum medium/floridanum*	Tolerant
C3 mostly graminoid; seed bank;	1	*Rhynchospora baldwinii*	Tolerant
not tall	1	*Rhynchospora chapmanii*	Tolerant
	1	*Rhynchospora oligantha*	Tolerant
	1	*Rhynchospora plumosa*	Tolerant
	1	*Scleria pauciflora*	Tolerant
	2	*Andropogon mohrii*	Tolerant
Fall flowering C4 grasses; tall;	2	*Andropogon virginicus*	Tolerant
deep roots; low leaf moisture;	2	*Anthaenantia rufa*	Tolerant
high specific leaf mass	2	*Aristida palustris*	Tolerant
	2	*Muhlenbergia expansa*	Tolerant
	2	*Schizachyrium tenerum*	Tolerant
	3	*Bigelowia nudata*	Tolerant
C3 medium height; scapose,	3	*Coreopsis linifolia*	Tolerant
deep mostly porous roots; fire-	3	*Lacnanthes caroliniana*	Tolerant
stimulated flowering; mostly	3	*Lophiola aurea*	Tolerant
high leaf moisture	3	*Triantha racemosa*	Tolerant
	3	*Xyris ambigua*	Tolerant
C3 tall, deep and porous roots;	4	*Helianthus heterophyllus*	
no fire-stimulated flowering	4	*Tiedemannia filiformis*	
Short, short-lived carnivorous;	5	*Drosera capillaris*	
non-porous shallow or no roots	5	*Utricularia subulata*	
C3 deep non-porous roots; fire-	6	*Balduina uniflora*	
stimulated flowering; scapose;	6	*Xyris baldwinii*	

Functional Group Defined by k-R Clustering	Functional Group Number	Species	Competitive Response Category
C3 shallow non-porous roots; no fire-stimulated flowering;	7	*Lachnocaulon anceps*	
	7	*Pinguicula planifolia*	
C3 shallow non-porous roots; no fire-stimulated flowering; scapose; not tall	7	*Rhynchospora latifolia*	
C3 thick rhizome or corm; deep roots; medium height, fire-	8	*Carphephorus pseudoliatris*	
	8	*Xyris caroliniana*	
Tall; summer flowering; thick rhizome	9	*Ctenium aromaticum*	
	9	*Rhexia alifanus*	
Club moss; prostrate; spore bank; shallow roots; not tall	10	*Lycopodiella alopecuroides*	Sensitive
	10	*Pseudolycopodiella caroliniana*	Sensitive
Long-lived carnivorous; thick rhizome; shallow non-porous	11	*Sarracenia alata*	Sensitive
	11	*Sarracenia psittacina*	Sensitive
Semi-woody; very low leaf moisture; very high specific leaf mass	12	*Hypericum brachyphyllum*	Sensitive
Orchid, thick corm, fire-stimulated flowering, vegetative	13	*Calopogon pallidus*	Sensitive
	13	*Pogonia ophioglossoides*	Sensitive
Short-lived, shallow and non-porous roots, not carnivorous, rhizomatous; not tall	14	*Scleria reticularis*	Sensitive
Polygala; short, not scapose; short-lived; fire-stimulated	15	*Polygala chapmanii*	Sensitive
	15	*Polygala cruciata*	Sensitive
C3 medium height, spring flowering, perennial, shallow roots	16	*Drosera tracyii*	Sensitive
	16	*Erigeron vernus*	Sensitive
	16	*Rhexia lutea*	Sensitive

Functional Group Defined by k-R Clustering	Functional Group Number	Species	Competitive Response Category
C3 thick rhizome or corm; mostly deep roots, some porous, some not; not tall; high leaf moisture	17	*Chaptalia tomentosa*	Sensitive
	17	*Eriocaulon compressum*	Sensitive
	17	*Eriocaulon decangulare*	Sensitive
	17	*Hypoxis wrightii*	Sensitive
	17	*Xyris drummondii*	Sensitive
	17	*Zigadenus glaberrimus*	Sensitive

Nomenclature: USDA Plants Database, except Tiedemannia filiformis (Walter) Feist & S.R. Downie and Dichanthelium ensifolium (Baldwin ex Elliott) Gould, which follow A Weakley (2018, Flora of Mississippi, working draft)
